# GeneXpert MTB/RIF Assay for the Diagnosis of Tuberculous Lymphadenitis on Concentrated Fine Needle Aspirates in High Tuberculosis Burden Settings

**DOI:** 10.1371/journal.pone.0137471

**Published:** 2015-09-14

**Authors:** Mulualem Tadesse, Gemeda Abebe, Ketema Abdissa, Dossegnaw Aragaw, Kedir Abdella, Alemayehu Bekele, Mesele Bezabih, Ludwig Apers, Bouke C. de Jong, Leen Rigouts

**Affiliations:** 1 Department of Medical Laboratory Sciences and Pathology, Jimma University, Jimma, Ethiopia; 2 Mycobacteriology Research Center, Institute of Biotechnology Research, Jimma University, Jimma, Ethiopia; 3 Department of Clinical Sciences, Institute of Tropical Medicine, Antwerp, Belgium; 4 Mycobacteriology Unit, Department of Microbiology, Institute of Tropical Medicine, Antwerp, Belgium; 5 Department of Biomedical Sciences, University of Antwerp, Antwerp, Belgium; University of Utah Health Sciences Center and ARUP Laboratories, UNITED STATES

## Abstract

**Introduction:**

The diagnosis of tuberculous lymphadenitis (TBL) remains challenging. The routinely used methods (cytology and smear microscopy) have sub-optimal sensitivity. Recently, WHO recommends GeneXpert to be used as the initial diagnostic test in patients suspected of having extra-pulmonary tuberculosis (EPTB). However, this was a conditional recommendation due to very low-quality evidence available and more studies are needed. In this study we evaluated the performance of Xpert for the diagnosis of TBL on concentrated fine needle aspirates (FNA) in Southwest Ethiopia.

**Methods:**

FNA was collected from presumptive TBL cases. Two smears were prepared from each aspirate and processed for cytology and conventional microscopy. The remaining aspirate was treated with N-acetyl-L-cysteine-NaOH and centrifuged for 15minutes at 3000g. The concentrated sediment was used for culture and Xpert test. Capilia TB-Neo test was used to differentiate *M*. *tuberculosis* complex (MTBC) from non-tuberculous mycobacteria (NTM). Composite bacteriological methods (culture and/or smear microscopy) were considered as a reference standard.

**Result:**

Out of 143 enrolled suspects, 64.3% (92/143) were confirmed TBL cases by the composite reference standard (CRS). Xpert detected *M*. *tuberculosis* complex (MTBC) in 60.1% (86/143) of the presumptive TBL cases. The sensitivity of Xpert compared to CRS was 87.8% [95% CI: 81.0–94.5] and specificity 91.1% [95% CI: 82.8–99.4]. The sensitivity was 27.8% for smear microscopy and 80% for cytology compared to CRS. Cytology showed the lowest specificity (57.8%). Xpert was positive in 4 out of 45 culture- and smear-negative cases. Among 47 cytomorphologically non-TBL cases, 15 were positive on Xpert. More than half of Xpert-positive cases were in the range of very low cut-off threshold values (28<Ct<38). Resistance to rifampicin was identified in 4.7% (4/86) of Xpert-positive cases.

**Conclusion:**

Xpert test showed a high sensitivity and specificity for the diagnosis of TBL on concentrated FNA samples. In addition, Xpert offered rapid detection of rifampicin-resistant *M*. *tuberculosis* strains from lymph node aspirates.

## Introduction

Tuberculosis (TB) remains a major public health problem in Ethiopia. Ethiopia ranks eighth in the list of the 22 high TB burden countries and 3^rd^ in terms of the number of extra-pulmonary tuberculosis (EPTB); of which 80% is localized in lymph nodes [1,2]. Accurate diagnosis and early treatment of TB has the potential to reduce morbidity and mortality associated with TB lymphadenitis (TBL). However, the differential diagnosis of TBL is broad and laboratory confirmation is of paramount importance to guide appropriate therapy [3,4].

Cytology and conventional smear microscopy have been used as the initial diagnostic tools for TBL in resource poor settings [4,5]. Fine needle aspiration cytology is a simple and rapid diagnostic technique, but with low specificity because of the presence of similar cytologic indicators in lesions other than those associated with TB [6,7]. Conventional smear microscopy lacks sensitivity due to the paucibacillary nature of fine needle aspirates (FNA) [8]. Mycobacteriological culture and drug susceptibility testing are not always available in resource poor settings like Ethiopia [1,9]. In line with these limitations more rapid and reliable methods are needed. In December 2010, WHO endorsed GeneXpert MTB/RIF® (Cepheid, USA) for use in TB laboratories. The Xpert assay consists of a closed system that is based on real-time polymerase chain reaction (PCR). It can be used by operators with minimal technical expertise, enabling the diagnosis of TB and simultaneous detection of rifampicin resistance within 2 hours [10].

The Xpert assay has been validated and optimized for sputum samples to diagnose HIV-associated TB and multidrug-resistant TB. WHO strongly recommends widespread use of Xpert for these groups of patients [11,12]. More recently a number of studies were done to evaluate this assay using non-respiratory clinical samples from patients suspected of having EPTB [9,13,14]. In 2014, WHO has recommended Xpert over the conventional tests (including conventional microscopy, culture or histopathology) for testing specific non-respiratory specimens (lymph nodes and other tissues) from patients suspected of having EPTB[15]. However, this was a conditional recommendation due to very low-quality evidence available. More studies are therefore needed particularly in settings with high EPTB prevalence. Thus, we evaluated the performance of Xpert for the diagnosis of TBL using routinely collected FNA samples and compared it against cytology, smear microscopy and culture.

## Materials and Methods

Ethical clearance was first obtained from the Jimma University ethical review board. A letter of permission to conduct the study was obtained from Jimma University Specialized Hospital clinical director office. All patients or guardians in case of children were requested for written consent prior to enrolment to the study. Any information concerning the patients was kept confidential. Laboratory results were reported back to the physicians for treatment initiation or decision as early as available.

This study was conducted at Jimma University Specialized Hospital, a public tertiary care hospital, in Southwest Ethiopia. A total of 143 consecutive outpatients clinically suspected of TBL and referred by attending clinicians for TB testing were enrolled in this study. Participants’ demographic and clinical information were collected using a pre-tested questionnaire. The FNA sample, at least 1ml, was collected by a pathologist in the pathology diagnostic unit. Gross specimen appearance (caseous, purulent, and/or blood stained) was recorded at the time of specimen collection. The first few drops of the aspirates were used for cytomorphological diagnosis. Air dried smears were stained with Wright’s stain and examined by a pathologist. The cytological criteria for the diagnosis of TBL are based on the presence of the following cytomorphological appearances: epithelioid cell aggregate with or without Langerhans giant cells and necrosis, epithelioid cell aggregate without necrosis, necrosis without epithelioid cell aggregate or polymorphonucleocytes with necrosis [16]. TB treatment was initiated based on the cytomorphological diagnosis.

The remaining sample was processed for smear microscopy, culture and Xpert in the Mycobacteriology Research Center at Jimma University. Two drops from each specimen were used to make a smear for standard Ziehl-Neelsen (ZN) staining. Stained smears were examined for the presence of AFB under oil-immersion (100x) using a light microscope. All AFB smear positive slides were graded based on the IUATLD scale [17].

Mycobacterial culture was done on Löwenstein-Jensen (LJ) medium within 2 days of specimen collection. All FNA specimens were processed by the standard *N*-acetyl-L-cysteine and sodium hydroxide (NALC/NaOH) method with a final NaOH concentration of 1% [17]. An equal volume of standard NALC-NALC/NaOH solution was added to the specimen and incubated for 15 minutes. After neutralization by phosphate buffered saline (PBS) and centrifugation (15 minutes at 3000g), the sediment was re-suspended in 1ml of sterile PBS. Finally 200μl of sediment was used to inoculate on two LJ slants. The laboratory strain, *M*. *tuberculosis* H_37_Rv (ATCC 27294), was used as a positive control. Random slants of LJ medium were inoculated with sterile distilled water in each run as negative controls. Culture positive results were confirmed for MTBC by Capilia TB-Neo test (TAUNS, Izunokuni, Japan).

Due to delay in transportation of Xpert cartridges, the remaining sediment was stored at -20°C. The median (IQR) delay before Xpert testing was 41 (30–45) days. Xpert test was performed using frozen and thawed sediment as previously described [18]. The sample reagent (1.5ml) supplied with the test was added in a 3:1 ratio to the sample sediment (0.5ml). The mixture was vortexed and incubated at room temperature for 15 minutes. Two ml of the reagent sample mix was then transferred to an Xpert cartridge using a pasteur pipette and the cartridge was loaded onto Xpert (Cepheid, Dx System Version 4.0c) machine. Results were reported as positive or negative for *M*. *tuberculosis*, including a semi-quantitative scale based on the quantitative cycle threshold (*Ct*) value of probe A. Rifampicin resistance results were reported as susceptible, resistant or indeterminate.

Data were double entered and analysed using the SPSS software package (version 16). Sensitivity, specificity, positive and negative predictive values with their corresponding 95% CIs were calculated using composite bacteriological methods (Culture for *M*. *tuberculosis* on LJ medium and/or smear microscopy using ZN method) as a reference standard. Study reporting and analysis were consistent with the standards for the reporting of diagnostic (STARD) accuracy studies checklist which is attached as supplementary information file (S1 Table).

## Results

**Fig 1:**
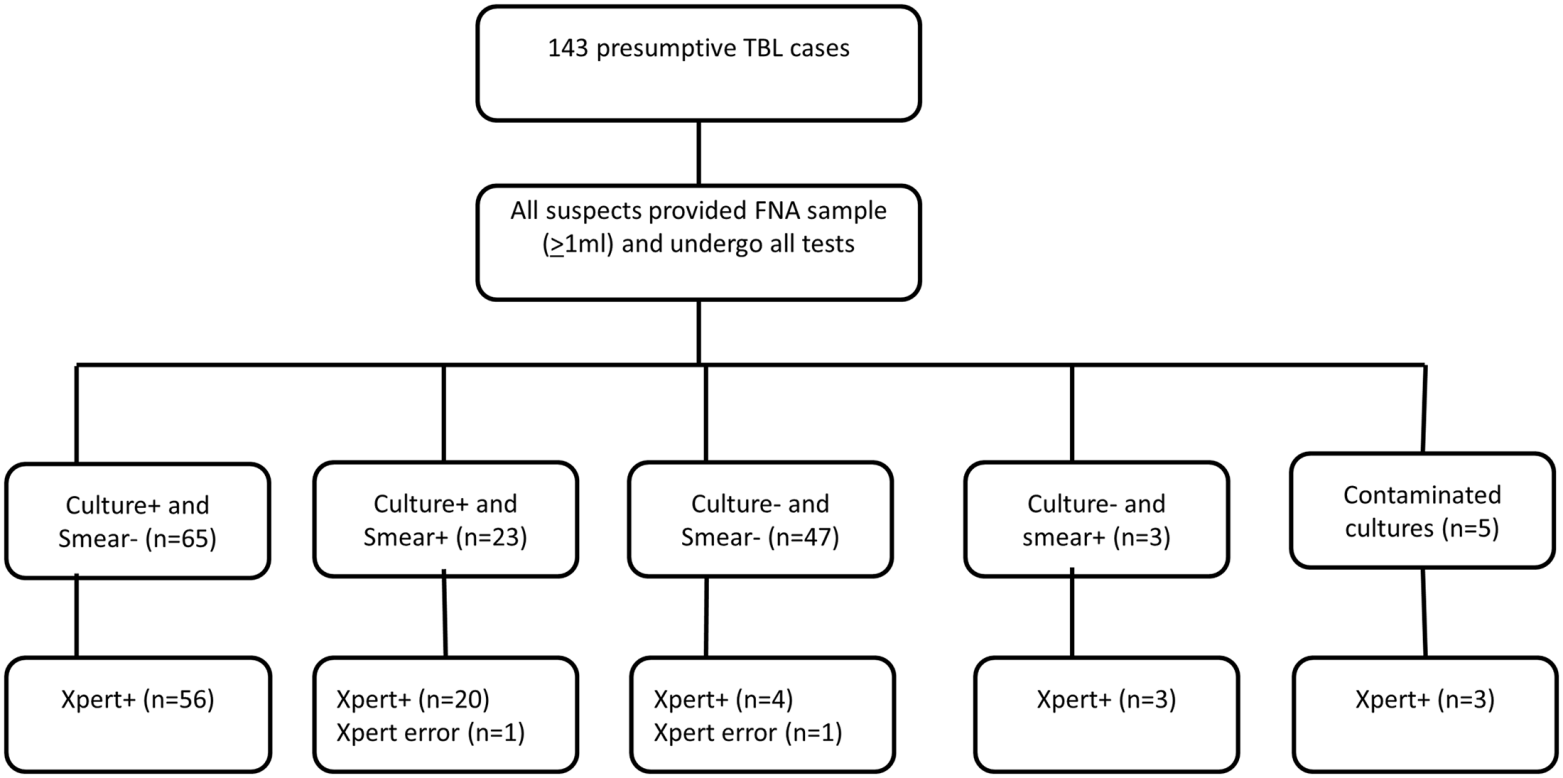
Diagnostic accuracy of Xpert for detection of *M*. *tuberculosis* from patients with suspected TBL. TBL = tuberculous lymphadenitis, FNA = fine needle aspirate, + = positive,— = negative.

A total of 143 patients with clinical presumptive TB presenting with lymphadenopathy were enrolled between May-September 2013. Out of these, 18.9% (27/143) were positive for TBL on smear microscopy, 60.1% (86/143) on Xpert and 61.5% (88/143) on culture. On cytological examination, 67.1% (96/143) had cytomorphological features suggesting TBL. Overall, 64.3% (92/143) of tested cases were positive for TBL by culture and/or smear microscopy (23 smear/culture-positive, 65 culture-positive/smear-negative, 3 smear-positive/culture-negative and 1 smear-positive/culture contaminated) (Fig 1). The Xpert result was invalid for 1.4% (2/143) of tests performed. Patients demographic and lymph node characteristics are shown in Table 1.

**Table 1:** Demographic and lymph node characteristics of TBL suspects included in this study (n = 143).

**Demographics characteristics**		**N (%)**
Sex	Male	67 (46.9)
	Female	76 (53.1)
Age-years	≤ 15	22 (15.4)
	16–30	83 (58.0)
	31–45	23 (16.1)
	>45	15 (10.5)
**Lymph node characteristics**		**N (%)**
Lymph node sites	Cervical	100 (69.9)
	Axillary	29 (20.3)
	Inguinal	14 (9.8)
Specimen appearance	Purulent	73 (50.1)
	Caseous	58 (40.6)
	Bloody stained	12 (8.4)

Smear microscopy detected AFB in 26% (23/88) of culture-positive and 6% (3/49) of culture-negative cases. Of five contaminated samples on culture, one was positive on smear microscopy. Culture was positive in 74% (71/96) of cases with suggestive cytomorphology of TB and in 36.2% (17/47) of non-TBL suggestive cases. When compared to CRS, smear microscopy had 27.8% sensitivity and 100% specificity whereas cytology showed sensitivity of 80.0% and specificity of 57.8%. Xpert was positive for *M*. *tuberculosis* in 86.4% (76/88) of culture-positive and 14.3% (7/49) of culture-negative cases. *M*. *tuberculosis* DNA was detected by Xpert in 3 out of 5 samples with contaminated cultures (Fig 1). Only one isolate was identified as NTM by the Capilia test and Xpert result was negative. MTBC isolates resistant to rifampicin were identified in 4.7% (4/86) of Xpert positive cases. Rifampicin resistance status for one MTBC positive sample (very low grade) was indeterminate.

Xpert showed an overall sensitivity of 87.8% and specificity of 91%. Xpert yielded a positive result in 56 out of 65 smear-negative/culture-positive cases. Only two specimens that were reported as “scanty AFB” in smear microscopy were negative by Xpert. When the culture positive results are stratified by AFB smear results, the sensitivity of Xpert was 91% (20/22) in smear-positive and 86.2% (56/65) in smear-negative cases. A summary of the diagnostic accuracy of cytology and Xpert test compared to CRS is presented in Table 2.

**Table 2:** Diagnostic accuracy of cytology and Xpert test as compared to composite reference standard (CRS).

Diagnostic accuracy	Cytology	Xpert
Sensitivity [95%CI]	80.0%[72.1–88.3]	87.8%[81.0–94.5]
Specificity [95%CI]	57.8%[43.3–72.2]	91.1%[82.8–99.4]
PPV [95%CI]	79.1%[70.7–87.5]	95.2%[90.5–99.8]
NPV [95%CI]	59.1%[44.6–73.6]	78.8%[67.7–89.9]
LR+ [95%CI]	1.9 [1.3–2.7]	9.8 [3.8–25.2]
LR- [95%CI]	0.3 [0.2–0.6]	0.1 [0.08–0.2]

PPV = positive predictive value, NPV = negative predictive value, CI = confidence interval, LR+ = likelihood ratio positive, LR- = likelihood ratio negative. The reference standard was culture for *M*. *tuberculosis* and/or smear microscopy for acid fast bacilli (AFB).

Xpert test assigns a semi-quantitative grade (very low, low, medium and high) on the basis of the Ct value to each test positive for *M*. *tuberculosis* and these categories seem to be predictive of the bacterial load [19]. Among 86 Xpert positive samples, 53.5% (46/86) were very low (28<Ct<38), 44.2% (38/86) low (22<Ct<28), and only 2 were in the ‘medium’ (16<Ct<22) category (Table 3). The mean Ct values were lower for smear-positive specimens compared to those smear-negative specimens (28.2 versus 31.1).

**Table 3:** Comparison of Xpert semi-quantitative result (Ct-value) and AFB smear grade.

	AFB smear grade				
Xpert result (Ct range)	Negative	Scanty	1+	2+	Total (N)
Very low (28<Ct≤38)	76% (35)	24%(11)	0	0	46
Low (22<Ct≤ 28)	71%(27)	5.3%(2)	23.7%(9)	0	38
Medium (16<Ct ≤ 22)	0	0	50.0% (1)	50.%(1)	2
High (Ct ≤ 16)	0	0	0	0	0

AFB = acid-fast bacilli; Ct = cycle threshold

The cytomorphological features consistent with TB were observed in 67% (96/143) of the patients with lymphadenitis. The other diagnoses reported on cytology were chronic inflammation in 11.2% (16/143), suppurative abscess in 10.5% (15/143), reactive lymphadenitis in 7.7% (11/143) and malignancy in 3.5% (5/143). Xpert detected *M*. *tuberculosis* in 74% (71/96) of cases with suggestive cytomorphology of TB. In addition, Xpert was also positive in 15 cases with negative cytology: 10 were from suppurative abscesses. In all these latter cases, TBL was confirmed by the CRS (Table 4).

**Table 4:** Comparisons of microscopic cytomorphological features and FNA gross appearance with Xpert test and CRS (culture and/or ZN).

		Xpert	CRS (culture and/or ZN)
FNA cytology result	Total (N)	Positive %(n/N)	Positive %(n/N)
TBL	96	74%(71/96)	77% (74/96)
Chronic inflammation	16	18.8%(3/16)	43.8%(7/16)
Suppurative abscess	15	66.7%(10/15)	66.7%(10/15)
Reactive lymphadenitis	11	9%(1/11)	0
Malignancy	5	20%(1/5)	20%(1/5)
**Gross FNA appearance**			
Purulent	73	56.2%(41/73)	60.3%(44/73)
Caseous	58	69%(40/58)	72.4%(42/58)
Bloody stained	12	41.7%(5/12)	50%(6/12)

TBL = tuberculous lymphadenitis, FNA = fine needle aspirate, CRS = composite reference standard, ZN = Ziehl-Neelsen.

Gross lymph node aspirate was described as purulent in 51% (73/143), caseous in 40.6% (58/143) and blood stained in 8.4% (12/143) of the cases. Xpert positivity rate was highest in aspirates with caseous appearance (69% (40/58)), and lowest in blood stained aspirates (41.7% (5/12)), although these differences were statistically not significant (Table 4).

## Discussion

The WHO and the Ethiopian national Implementation Guideline for GeneXpert strongly recommend the use of Xpert for the initial diagnosis of individuals suspected of MDR-TB or HIV associated TB [12]. Based on very low quality evidence, WHO also conditionally recommends Xpert to be used rather than conventional methods as the initial diagnostic test in patients suspected of having EPTB [12,15]. In Southwest Ethiopia, where TB and MDR-TB are highly prevalent, the effectiveness of Xpert for diagnosing TBL and/or detection of drug resistance has not been conclusively demonstrated.

In the present study, the sensitivity of Xpert was 87.8%. A systematic review and meta-analyses conducted by Denkinger *et al* showed that Xpert test has a sensitivity ranging from 50% to 100% with pooled sensitivity of 83%[20] More recently, Penz *et al* reviewed 36 studies in their meta-analyses and confirmed Xpert pooled sensitivity of 87% that is similar to our study[21]. However, the sensitivity of Xpert in the current study is lower than what was found in similar study by Ligthelm *et al* (sensitivity, 96.7%) [22]. There were 11 culture-positive cases which were negative on Xpert. The reason for false-negative Xpert test results may be due to the limited number of bacilli in the FNA sample or prolonged storage (median (IQR) delay of 41 (30–45) days) of sample before Xpert testing.

The specificity (91%) of the Xpert in the current study was found to be consistent with previous studies reported by others (specificity, 89–99%) [20,21,22], but higher than the study done by Biadigilegn *et al* (specificity, 69.2%) [13]. Seven culture-negative cases were Xpert-positive. Five of these were positive for TBL on cytology and 3 on smear microscopy, suggesting the presence of nonviable bacilli due to either the harsh decontamination process or the nature of the caseous lesion in the lymph node tissue which may have contained dead tubercle bacilli. Such cases (Xpert-positive but culture-negative) are likely to be true TB positives as corroborated by the high specificity [14,18] and by the fact that the procedure is less prone to contamination due to the closed reaction chamber (real-time PCR technology) of Xpert.

Even though conventional ZN microscopy has played an important role in the diagnosis of TBL in resource poor settings, Xpert detected MTB in 86% (56/65) of cases missed by smear microscopy. Only 2 smear-and culture-positive samples were negative by Xpert. In agreement with other studies [9,18,22], Xpert has a higher sensitivity than smear microscopy. There was little difference in the sensitivity of Xpert in smear-positive and smear-negative TBL cases. Xpert detected a significant proportion of smear-negative and culture-positive cases and significantly increased the relative proportion of diagnosed TBL cases.

In developing countries, smear microscopy is the only widely implemented method for quantifying the bacterial burden at the time of the initial diagnosis [23]. Xpert provides a semi-quantitative measurement of the number of MTBC bacilli present in a clinical sample. In this study, more than 90% of Xpert-positive samples were scored as ‘low’ and ‘very low’ suggesting a limited number of bacilli in FNA sample.

FNA cytology as an inexpensive and reliable tool for TBL has been studied by a number of investigators [4,24,25]. It is one of the most commonly used methods in resource poor settings. In the current study the sensitivity of cytology was comparable to that of Xpert, but the specificity was lower (57.8%), yielding many false positives. This may be due to non-specific cytomorphological features seen in cytology. On the other hand, cytomorphological features associated with suppurative abscess did not reliably exclude TBL in our study, which may be explained by the absence of characteristic features such as scattered epithelioid cells among the polymorphous population of lymphoid cells- indeed, our findings suggest that ‘suppurative’ features should be considered as suggestive of TB as the cause of the lymphadenitis.

To the best of our knowledge, no information regarding the drug resistance pattern of mycobacterial strains isolated from TBL patients in Southwest Ethiopia is available. Xpert test offers rapid detection of rifampicin resistant MTBC strains directly from the clinical sample, an important advantage over cytology and smear microscopy. Previous studies reported 98–100% agreement in detection of rifampicin resistance strains using the Xpert test and phenotypic drug susceptibility test [13,14,18,26]. In the current study, rifampicin resistance was identified in 4.7% (4/86) of Xpert-positive cases. Two of these were retreatment cases.

Our study has some limitations. Mycobacterial culture on LJ medium and/or smear microscopy was used as a reference standard though both of these methods are not sufficient to detect all TBL cases. Among culture and/or smear-negative cases there may be false negatives that started anti-TB treatment on clinical grounds and improved, cases that were most likely true TB. Unfortunately we did not include clinical outcomes in our data set. Thus, further prospective studies are required to evaluate the performance of Xpert on unprocessed fresh FNA samples by using a more sensitive liquid culture and/or histology as a reference standard or by adding clinical diagnosis (with response to treatment) to the standards. Moreover, while we only identified one NTM in culture, we did not speciate it, and our study is unable to reflect on the contribution in Ethiopia of NTMs known to cause lymphadenitis, such as *M*. *scrofulaceum*, *M*. *avium* complex, and *M*. *kansasii*.

In conclusion, our findings indicate that Xpert MTB/RIF test is a useful tool for the detection of MTBC with high sensitivity and specificity on concentrated fine needle aspirate with superior performance as compared to cytology and smear microscopy. Besides improved sensitivity, the Xpert was able to identify patients with TBL due to rifampicin resistant TB. The Xpert test is an easy and suitable method to be used in TB endemic settings and its implementation could significantly improve the rapid diagnosis of TBL.

## Supporting Information

S1 Fig Overall study flowchart explaining the patient recruitment, sample processing and diagnostic test results. (DOCX)

S1 Table STARD checklist completed for manuscript on GeneXpert MTB/RIF assay for the Diagnosis of Tuberculous Lymphadenitis on Concentrated Fine Needle Aspirates in High Tuberculosis Burden Settings. (DOCX)

S2 Table Xpert test result compared to composite reference standard for the diagnosis of TBL in 135 lymph node aspirates. (DOCX)

S3 Table FNA cytology result compared to composite reference standard for the diagnosis of TBL. (DOCX)

S4 Table Distribution of Xpert cycle threshold (Ct) values according to AFB smear grade. (DOCX)
