# Supplementary material for: GeneXpert MTB/RIF Assay for the Diagnosis of Tuberculous Lymphadenitis on Concentrated Fine Needle Aspirates in High Tuberculosis Burden Settings
Source: PLoS One. 2015 Sep 14;10(9):e0137471. doi: 10.1371/journal.pone.0137471 (PMC4569183; doi:10.1371/journal.pone.0137471)
Supplement: S1 Fig — (DOCX) [file pone.0137471.s001.docx]

**Overall study flowchart**

143 consecutive patients referred by physicians for TB testing were included.

Inclusion criteria:

- Presumptive case of TBL
- Consented to provide FNA (> 1ml)

FNA specimen

Smear microscopy (ZN)

FNA cytology

NALC-NaOH

Culture (LJ medium)

Sample sediments were stored at -20^0^C

Xpert MTB/RIF test

Preliminary data analysis (N=143)

Culture (LJ)

- Positive = 88

- Negative= 50

- NTM = 1

-Contaminated=5

FNA cytology result

- Consistent with TBL= 96

- Chronic inflammation= 16

- Suppurative abscess= 15

- Reactive lymphadenitis= 11

- Malignancy = 5

Xpert MTB/RIF

- Positive = 86

- Negative= 55

- Invalid = 2

Smear microscopy

- Positive = 27

- Negative= 116

8 were excluded

- 5 contaminated cultures

-2 Xpert invalid results

- 1 NTM isolate on culture

135 were included in the diagnostic test accuracy calculation

CRS positive (n= 90)

CRS negative (n= 45)

Smear positive = 25

Cytology suggestive of TB= 72

Xpert positive = 79

Smear positive = 0

Cytology suggestive of TB= 19

Xpert positive = 4

**Supplementary figure-1: Flowchart explaining the patient recruitment, sample processing and diagnostic test results.** TBL=TB lymphadenitis, FNA=fine needle aspirate, NALC=*N*-acetyl-L-cysteine, NTM= non-tuberculous mycobacteria, CRS=composite reference standard.
