# Supplementary material for: GeneXpert MTB/RIF Assay for the Diagnosis of Tuberculous Lymphadenitis on Concentrated Fine Needle Aspirates in High Tuberculosis Burden Settings
Source: PLoS One. 2015 Sep 14;10(9):e0137471. doi: 10.1371/journal.pone.0137471 (PMC4569183; doi:10.1371/journal.pone.0137471)
Supplement: S1 Table — (DOCX) [file pone.0137471.s002.docx]

**Supplementary Table 1.** STARD checklist completed for manuscript on GeneXpert MTB/RIF assay for the Diagnosis of Tuberculous Lymphadenitis on Concentrated Fine Needle Aspirates in High Tuberculosis Burden Settings.

| **Section and Topic** | **Item** |  | **On page** |
| --- | --- | --- | --- |
| TITLE/ABSTRACT/ KEYWORDS | 1 | To evaluate the performance of Xpert for the diagnosis of tuberculous lymphadenitis (TBL) on concentrated fine needle aspirates (FNA) in high TB burdened settings. | 1 |
| INTRODUCTION | 2 | WHO strongly recommends widespread use of Xpert for the diagnosis of HIV-associated TB and MDR-TB ([11](#_ENREF_11)). More recently a number of studies were done to evaluate Xpert assay using non-respiratory clinical samples ([9](#_ENREF_9), [12](#_ENREF_12), [13](#_ENREF_13)) . However, the available data are insufficient to make an evidence based recommendation towards Xpert implementation as a primary diagnostic tool and more studies are warranted. In this study we evaluate the performance of Xpert for the diagnosis of TBL using routinely collected lymph node aspirate and compare it against cytology, smear microscopy and culture. | 3 |
| METHODS |  |  |  |
| *Participants* | 3 | A prospective study was carried out at Jimma University Specialized Hospital, a public tertiary care hospital, in Southwest Ethiopia. Adult consecutive patients with enlarged lymph nodes, clinically suspected for TBL, and consented to provide fine needle aspirate (FNA) were enrolled in this study. Patients on anti-tuberculosis treatment at the time of the lymph node aspiration were excluded from the study. | 4 |
|  | 4 | [All consecutive patients with clinical presumptive of TBL by attending clinician and subjected for FNA were recruited.](http://www.stard-statement.org/item4_maintext.htm) | 4 |
|  | 5 | A consecutive series of patients presenting at the pathology diagnostic unit were prospectively enrolled during the study period. | 4 |
|  | 6 | We prospectively collected data from 143 TBL suspected patients. Fine needle aspirate (FNA) was collected by a pathologist and processed for cytology, smear microscopy and culture. The remaining sample was stored at -20^0^C and tested for Xpert assay. | 4 & 5 |
| *Test methods* | 7 | Composite bacteriological methods (culture and/or smear microscopy) were considered as a reference standard. Even though culture has reduced sensitivity in EPTB cases, it still remains superior to other diagnostic modalities. Combined use of culture and smear microscopy may further increases the sensitivity. Several published articles including those cited in this manuscript considered culture as the reference standard method. | 5 |
|  | 8 | On spot of specimen collection, a smear was prepared for cytomorphological diagnosis. Air dried smears were stained with Wright’s stain and examined by a pathologist under microscopy ([14](#_ENREF_14)). The remaining sample was processed for smear microscopy on the same day. Two drops from each specimen were used to make a smear for standard Ziehl-Neelsen (ZN) staining. Stained smears were examined for the presence of AFB under oil-immersion (100x) using a light microscope. Mycobacterial culture was done on Löwenstein-Jensen (LJ) medium within 2 days of specimen collection (samples were kept at 4^0^C until processing). All FNA specimens were processed by the standard *N*-acetyl-L-cysteine and sodium hydroxide (NALC/NaOH) method with a final NaOH concentration of 1% ([15](#_ENREF_15)). An equal volume of standard NALC-NALC/NaOH solution was added to the specimen and incubated for 15 minutes. After neutralization by phosphate buffered saline (PBS) and centrifugation (15 minutes at 3000g), the sediment was re-suspended in 1ml of sterile PBS. Finally 200µl of sediment was used to inoculate on two LJ slants. Culture positive results were confirmed for MTBC by Capilia TB-Neo test (TAUNS, Izunokuni, Japan). Xpert test was performed using frozen and thawed sediment as previously described ([16](#_ENREF_16)). The sample reagent (1.5ml) supplied with the test was added in a 3:1 ratio to the sample sediment (0.5ml). The mixture was vortexed and incubated at room temperature for 15 minutes. Two ml of the reagent sample mix was then transferred to an Xpert cartridge using a pasteur pipette and the cartridge was loaded onto Xpert (Cepheid, Dx System Version 4.0c) machine. | 4 & 5 |
|  | 9 | The cytomorphological criteria for the diagnosis of TBL are based on the presence of the following cytomorphological appearances: epithelioid cell aggregate with or without Langerhans giant cells and necrosis, epithelioid cell aggregate without necrosis, necrosis without epithelioid cell aggregate or polymorphonucleocytes with necrosis. Smear microscopy was considered positive if >1AFB/hpf was observed under light microscope. Culture results were reported as negative if no growth was observed within 8 weeks of inoculation, positive if >1 colonies of *M. tuberculosis* was identified, or contaminated if the growth of the micro-organisms other than *M. tuberculosis* was confirmed. Xpert results were reported as positive, negative or invalid for *M. tuberculosis.* Positive results were placed in one of four categories; very low, low, medium, or high depending on the quantitative cycle threshold (*Ct*) value of probe A. Rifampicin resistance results were reported as susceptible, resistant or indeterminate. | 4 & 5 |
|  | 10 | Cytomorphological examination was performed by two senior pathologists. Three specialist microbiologists who had four years experiences in TB laboratory carried out mycobacterial culture and identifications. Xpert assay consists of a closed system that automates most of the steps and can be used by operators with minimal technical expertise. Though a trained person involved in performing Xpert tests in this study. | 4 & 5 |
|  | 11 | The person performing Xpert were blinded to the patients’ clinical information and to results of reference standard (culture and/smear microscopy) and cytological findings. Similarly people reading standard culture results were masked for the results of other tests or even for all information related to the patients. Only pathologists reading FNA smear were get informed to patients’ clinical history, but they were still blinded to other test results. | 4 & 5 |
| *Statistical methods* | 12 | Sensitivity, specificity, positive and negative predictive values with their corresponding 95% CIs were calculated using composite bacteriological methods as a reference standard. | 5 |
|  | 13 | We could not evaluate the reproducibility of the test methods used in this study. Neither the index tests nor the reference standards may be adversely affected by inter-observer variability. |  |
| RESULTS |  |  |  |
| *Participants* | 14 | We prospectively collected and examined FNA sample from patients at pathology diagnostic unit of Jimma University, tertiary care university hospital, between May-September 2013. | 5 |
|  | 15 | **Table-1: Demographic and lymph node characteristics of TBL suspects included in this study (n=143).**   \| **Demographics characteristics** \|  \| **N (%)** \| \| --- \| --- \| --- \| \| Sex \| Male \| 67 (46.9) \| \|  \| Female \| 76 (53.1) \| \| Age-years \| < 15 \| 22 (15.4) \| \| 16-30 \| 83 (58.0) \| \| 31-45 \| 23 (16.1) \| \| >45 \| 15 (10.5) \| \| **Lymph node characteristics** \|  \| **N (%)** \| \| Lymph node sites \| Cervical \| 100 (69.9) \| \| Axillary \| 29 (20.3) \| \| Inguinal \| 14 (9.8) \| \| Specimen appearance \| Purulent \| 73 (50.1) \| \| Caseous \| 58 (40.6) \| \| Bloody stained \| 12 (8.4) \| |  |
|  | 16 | 143 presumptive TBL cases  All suspects provided FNA sample (>1ml) and undergo all tests  Contaminated culture (n=5)  Culture- and smear+ (n=3)  Culture- and Smear- (n=47)  Culture+ and Smear+ (n=23)  Culture+ and Smear- (n=65)  Xpert+ (n=3)  Xpert+ (n=20)  Xpert error (n=1)  Xpert+ (n=4)  Xpert error (n=1)  Xpert+ (n=56)  Xpert+ (n=3)  **Figure-1: Flowchart for GeneXpert diagnostic accuracy study.** |  |
| *Test results* | 17 | Patients were subjected for cytological examinations followed by smear microscopy on the spot of sample collections. Culture was performed within 2 days of sample collection while Xpert tests were performed from frozen sample after a median (IQR) delay of 41 (30-45) days. | 5 |
|  | 18 | [Based](http://www.stard-statement.org/item18_maintext.htm) on cytology result, 67% (96/143) of study participants were classified as TBL cases. The other diagnoses reported were chronic inflammation in 11.2% (16/143), suppurative abscess in 10.5% (15/143), reactive lymphadenitis in 7.7% (11/143) and malignancy in 3.5% (5/143). Xpert yielded a positive result in 74 and 66.7 percent of TBL and suppurative abscess cases respectively. Xpert positivity rate was highest in aspirates with caseous appearance (69% (40/58)), and lowest in blood stained aspirates (41.7% (5/12)), although these differences were statistically not significant. |  |
|  | 19 | **Xpert test result versus composite reference standard for the diagnosis of TBL in 135 lymph node aspirates.**   \|  \|  \| **Reference standard*** \| \|  \| \| --- \| --- \| --- \| --- \| --- \| \|  \|  \| Positive \| Negative \| Total \| \| **Xpert test** \| Positive \| 79 \| 4 \| 83 \| \| Negative \| 11 \| 41 \| 52 \| \|  \| Total \| 90 \| 45 \| 135 \|   *The reference standard was culture for *M. tuberculosis* and/or smear microscopy for acid fast bacilli (AFB). |  |
|  | 20 | [No adverse event while performing any](http://www.stard-statement.org/item20_maintext.htm) of the tests included in this study. Occasionally a small bleeding and some discomforts/pains were observed during collection of fine needle aspirates which can be taken care of by the clinicians. |  |
| *Estimates* | 21 | Diagnostic accuracy of cytology and Xpert test as compared to composite reference standard (culture and/or smear microscopy) are given in **Table-2.** The sensitivity and specificity (with 95% confidence interval) were 87.8% [81.0 - 94.5] and 91.1% [82.8- 99.4] for Xpert and 80% [72.1 - 88.3] and 57.8% [43.3 - 72.2] for cytology respectively. |  |
|  | 22 | Xpert invalid test results (not clearly positive or negative) were observed only in 1.4% (2/143) of tests performed. Contaminated cultures and invalid Xpert results were excluded from the sensitivity and specificity calculations. |  |
|  | 23 | The diagnostic accuracy and utility of the Xpert may vary between subgroups of participants (e.g. HIV status). Unfortunately we were unable to determine the diagnostic accuracy among subgroups. But we determined the sensitivity of Xpert in smear negative and positive specimens. When the culture positive results are stratified by AFB smear results, the sensitivity of Xpert was 91% (20/22) in smear-positive and 86.2% (56/65) in smear-negative. |  |
|  | 24 | Estimates of test reproducibility were not done. |  |
| DISCUSSION | 25 | More recently a number of studies were done to evaluate Xpert assay using non-respiratory clinical, though the available data are insufficient to make an evidence based recommendation ([9](#_ENREF_9), [12](#_ENREF_12), [13](#_ENREF_13)). In Southwest Ethiopia, the effectiveness of Xpert for diagnosing TBL and/or detection of drug resistance has not been conclusively demonstrated. In this study, we evaluated the performance of Xpert for the diagnosis of TBL using routinely collected FNA samples. These data suggest that Xpert MTB/RIF test is a useful tool for the detection of MTBC with high sensitivity and specificity on concentrated fine needle aspirate with superior performance as compared to cytology and smear microscopy. Xpert test also offers rapid detection of rifampicin resistant MTBC strains directly from the clinical sample, an important advantage over cytology and smear microscopy. We suspect, Xpert assay would perform well in our settings and its implementation could significantly improve the rapid diagnosis of TBL. |  |
