# Supplementary material for: GeneXpert MTB/RIF Assay for the Diagnosis of Tuberculous Lymphadenitis on Concentrated Fine Needle Aspirates in High Tuberculosis Burden Settings
Source: PLoS One. 2015 Sep 14;10(9):e0137471. doi: 10.1371/journal.pone.0137471 (PMC4569183; doi:10.1371/journal.pone.0137471)
Supplement: S2 Table — (DOCX) [file pone.0137471.s003.docx]

**Supplementary table-2: Xpert test result compared to composite reference standard for the diagnosis of TBL in 135 lymph node aspirates.**

|  |  | **Reference standard*** | |  |
| --- | --- | --- | --- | --- |
|  |  | Positive | Negative | Total |
| **Xpert test** | Positive | 79 | 4 | 83 |
|  | Negative | 11 | 41 | 52 |
|  | Total | 90 | 45 | 135 |

*The reference standard was culture for *M. tuberculosis* and/or smear microscopy for acid fast bacilli (AFB).
