# Supplementary material for: GeneXpert MTB/RIF Assay for the Diagnosis of Tuberculous Lymphadenitis on Concentrated Fine Needle Aspirates in High Tuberculosis Burden Settings
Source: PLoS One. 2015 Sep 14;10(9):e0137471. doi: 10.1371/journal.pone.0137471 (PMC4569183; doi:10.1371/journal.pone.0137471)
Supplement: S3 Table — (DOCX) [file pone.0137471.s004.docx]

**Supplementary table-3: FNA cytology result compared to composite reference standard for the diagnosis of TBL.**

|  |  | **Reference standard*** | |  |
| --- | --- | --- | --- | --- |
|  |  | Positive | Negative | Total |
| **FNA cytology** | TBL | 72 | 19 | 91 |
|  | Other diagnosis | 18 | 26 | 44 |
|  | Total | 90 | 45 | 135 |

*The reference standard was culture for *M. tuberculosis* and/or smear microscopy for acid fast bacilli (AFB). FNA=fine needle aspirate, TBL=tuberculous lymphadenitis.
