# Supplementary material for: GeneXpert MTB/RIF Assay for the Diagnosis of Tuberculous Lymphadenitis on Concentrated Fine Needle Aspirates in High Tuberculosis Burden Settings
Source: PLoS One. 2015 Sep 14;10(9):e0137471. doi: 10.1371/journal.pone.0137471 (PMC4569183; doi:10.1371/journal.pone.0137471)
Supplement: S4 Table — (DOCX) [file pone.0137471.s005.docx]

**Supplementary table 4: Distribution of Xpert cycle threshold (Ct) values according to AFB smear grade (n=86 Xpert positive specimens).**

| **Sample code** | **Smear grade** | **Ct-value** | **Sample code** | **Smear grade** | **Ct-value** | **Sample code** | **Smear grade** | **Ct-value** |
| --- | --- | --- | --- | --- | --- | --- | --- | --- |
| 893 | Negative | 25 | 1022 | Negative | 30 | 1084 | (1+) | 26 |
| 932 | Negative | 26 | 1026 | Scanty | 32 | 1085 | Negative | 31 |
| 939 | Scanty | 30 | 1027 | Negative | 32 | 1086 | Negative | 27 |
| 946 | Negative | 28 | 1030 | Negative | 26 | 1087 | Negative | 29 |
| 947 | Negative | 27 | 1033 | Negative | 31 | 1088 | Negative | 27 |
| 948 | (1+) | 28 | 1034 | Negative | 32 | 1089 | Negative | 27 |
| 954 | Negative | 35 | 1036 | Negative | 27 | 1090 | Negative | 28 |
| 973 | Negative | 32 | 1040 | Negative | 31 | 1092 | Negative | 26 |
| 979 | Scanty | 29 | 1041 | Negative | 32 | 1093 | Scanty | 28 |
| 982 | Negative | 33 | 1042 | Negative | 28 | 1094 | Negative | 27 |
| 985 | Negative | 29 | 1043 | (1+) | 22 | 1096 | (1+) | 28 |
| 986 | Scanty | 29 | 1044 | Scanty | 30 | 1099 | Negative | 32 |
| 987 | Negative | 27 | 1052 | Negative | 27 | 1101 | Negative | 29 |
| 988 | Negative | 26 | 1053 | Negative | 28 | 1104 | Negative | 26 |
| 989 | Negative | 32 | 1056 | (1+) | 27 | 1105 | Negative | 28 |
| 997 | Negative | 34 | 1057 | Negative | 32 | 1106 | Negative | 32 |
| 998 | (1+) | 25 | 1065 | Negative | 33 | 1107 | Negative | 31 |
| 999 | Scanty | 33 | 1067 | Negative | 32 | 1120 | Negative | 33 |
| 1002 | Scanty | 30 | 1069 | Negative | 34 | 1124 | Negative | 34 |
| 1003 | Negative | 28 | 1070 | Negative | 27 | 1125 | Negative | 32 |
| 1004 | (1+) | 26 | 1071 | Negative | 31 | 1131 | Scanty | 31 |
| 1005 | Negative | 30 | 1072 | Negative | 24 | 1132 | Negative | 35 |
| 1006 | Negative | 35 | 1077 | Negative | 27 | 1133 | Negative | 28 |
| 1007 | (1+) | 26 | 1078 | Scanty | 29 | 1134 | Negative | 32 |
| 1010 | Negative | 32 | 1079 | (2+) | 18 | 1137 | Negative | 27 |
| 1014 | Scanty | 33 | 1080 | Negative | 28 | 1138 | Negative | 31 |
| 1016 | Scanty | 34 | 1081 | Negative | 27 | 1139 | (1+) | 27 |
| 1020 | Negative | 34 | 1082 | Negative | 31 | 1140 | (1+) | 28 |
| 1021 | Scanty | 28 | 1083 | Negative | 31 |  |  |  |
